# Obesity—a key driver in osteoarthritis

**DOI:** 10.1210/jendso/bvag245

**Published:** 2026-10-22

**Authors:** Khaled Elhady Mohamed, Christian S Thudium, Asger Reinstrup Bihlet, Mette Sørensen Iken, Morten Asser Karsdal, Kim Henriksen, Anne-Christine Bay-Jensen

**Affiliations:** Nordic Bioscience, 2730 Herlev, Denmark; Department of Drug Design and Pharmacology, University of Copenhagen, 2100 Copenhagen, Denmark; Nordic Bioscience, 2730 Herlev, Denmark; NBCD, 2860 Soeborg, Denmark; Nordic Bioscience, 2730 Herlev, Denmark; Nordic Bioscience, 2730 Herlev, Denmark; KeyBioscience AG, 6370 Stans, Switzerland; Nordic Bioscience, 2730 Herlev, Denmark; KeyBioscience AG, 6370 Stans, Switzerland; Department of Molecular and Medical Biology, Roskilde University Center, 4000 Roskilde, Denmark; Nordic Bioscience, 2730 Herlev, Denmark

**Keywords:** obesity, weight loss, metabolic disease, osteoarthritis, pain, function

## Abstract

**Purpose:**

Obesity causes mechanical stress and metabolic dysfunction. However, the relationship between obesity and osteoarthritis (OA) pain still needs further investigation. This study aims to examine the effect of weight loss on pain and function through a secondary analysis of a randomized, double-blind, multicenter OA trial and to evaluate obesity's impact on OA pain using a rat model.

**Methods:**

In a post hoc analysis of a randomized controlled trial (RCT; NCT00486434), we evaluated baseline body mass index and Western Ontario and McMaster Universities Osteoarthritis (WOMAC) scores (*n* = 1175) and examined the impact of 5% weight loss on WOMAC scores after 2 years using a factorial analysis of covariance (*n* = 674). In a preclinical model of metabolic OA induced by meniscectomy, pain-like behavior was assessed with the von Frey and cold plate tests.

**Results:**

Participants who lost >5% of weight within 2 years experienced a mean decrease of −567.1 (95% CI −666.7 to −467.6) and −399.2 (−505.8 to −292.5) in total WOMAC scores for their target and nontarget knees, respectively, whereas individuals who gained >5% in weight had a smaller decrease of −364.6 (−496.5 to −232.7) and −135 (−277.4 to −6.3), respectively. Additionally, the high-fat diet–fed rat model showed more pain-like behavior than the lean model.

**Main conclusion:**

These studies indicate that weight loss could decrease OA pain in both target and nontarget knees of patients with obesity, supporting the inclusion of obesity in OA animal models to improve the translatability of OA treatments.

Obesity is one of the main health challenges facing the global population (1). Importantly, while obesity is the cause of many of the problems, the comorbidities are what impact the healthcare systems and associated costs (1). A major comorbidity of obesity is osteoarthritis (OA), which is characterized by structural deterioration of the affected joints and is accompanied by pain and loss of function, causing reduced quality of life (2, 3). Many studies have looked into the relationship between increased body mass index (BMI) and OA and have found that increased body weight is positively associated with a higher incidence of OA (1). While the mechanical stress from excessive weight is a major culprit in OA, as illustrated by the strong relationship between knee pain and obesity in OA (4-6). The correlation goes beyond just mechanical stress. This is illustrated by the increased incidence of hand OA in people with obesity (5). Although the relationship between pain in non–weight-bearing joints and obesity is not clear, there is evidence supporting that it is at least partially driven by systemic inflammation (7). Adipose tissues are known to release a range of adipokines, proinflammatory cytokines, and other mediators, such as leptin, interleukin-6, and C-reactive protein, which are some of the factors that may explain why patients with obesity tend to have lower pain thresholds compared to those without (7-9).

While the clinical data are quite consistent in showing a tight relationship between increased fat mass and OA (10), most OA animal models have focused on OA induction and disease progression, leaving out the influence of obesity and thereby forgoing this key interaction and possibly missing potential treatment targets. Hence, many unanswered questions remain regarding the interaction between obesity and joint pain. The underrepresentation of metabolically driven OA in preclinical models likely contributes to the difficulty of developing an effective drug for OA.

This post hoc study aimed to investigate the association between obesity, pain, and function in patients with confirmed OA enrolled in a phase III OA study who had been screened and carefully examined for pain and body weight. Furthermore, to investigate the translatability of the relationship preclinically, we examined a rat OA model fed a high-fat diet (HFD), assessing the potential of obesity as a driver of painful OA.

## Materials and methods

### Study population

#### Ethical approval

This post hoc study includes data from a double-blinded, randomized, placebo-controlled, and multicenter phase III clinical trial assessing the efficacy and safety of an oral formulation of 0.8 mg salmon calcitonin in patients with painful and radiographic knee OA (CSMC021C2301, https://clinicaltrials.gov/study/NCT00486434) completed September, 2009 (11). The trial was conducted in accordance with the Declaration of Helsinki and International Council for Harmonisation Good Clinical Practice (ICH GCP) and was approved by all applicable independent review boards, ethics committees, and regulatory bodies, Denmark 2612-2951, Czech Republic 12827/05, Estonia 07-022, Hong Kong PRCT322/2007, Lithuania 2005-004145, Poland CEBK/0292/07, and Romania 010494/15.10.2007.

#### Inclusion criteria

Regarding the inclusion criteria for the original study, the patients included met the OA diagnostic criteria set by the American College of Rheumatology (12). A target knee was selected before randomization to assess the primary endpoint of efficacy, as both knees were assessed throughout the study. The target knee must have exhibited pain for most of the previous month. Furthermore, the patient had to satisfy one more criterion from the following ones: older than 50 years, experiencing knee crepitus, or morning stiffness of fewer than 30 minutes. The medial tibiofemoral joint space width of the target knee measured by X-ray must be ≥2.0 mm, and a Kellgren and Lawrence (KL) score of 2 or 3 (13). Patients also needed to have a score of at least 150 mm for pain subscale (0-500 mm) or 510 mm for function subscale (0-1700 mm) on the Western Ontario and McMaster Universities Osteoarthritis (WOMAC) index (11).

The original study excluded patients who received intra-articular corticosteroid or hyaluronic acid injections in the signal knee within the study period or within 3 months before randomization. Patients who were diagnosed with diseases or were taking medications that affected bone or cartilage metabolism were excluded from the study. Pain relief medication was allowed 30 minutes after the original study drug administration (11).

#### Outcomes

Version VA3.1 of the WOMAC questionnaire (14): Patients answered questions assessing their pain, physical function, and stiffness. The questionnaire included 5 questions for the pain scale, 17 for the function scale, and 2 for stiffness. Each question had a scale of 0 to 100. Pain, physical function, and stiffness ranged from 0 to 500, 0 to 1700, and 0 to 200, respectively (11). This study included the baseline and final 2-year follow-up WOMAC scores. Reporting followed the CONSORT 2025 guidelines. Patients included in the post hoc analysis at baseline and after 2-year follow-up are depicted in the study flow diagram (Fig. 1).

**Figure 1:**
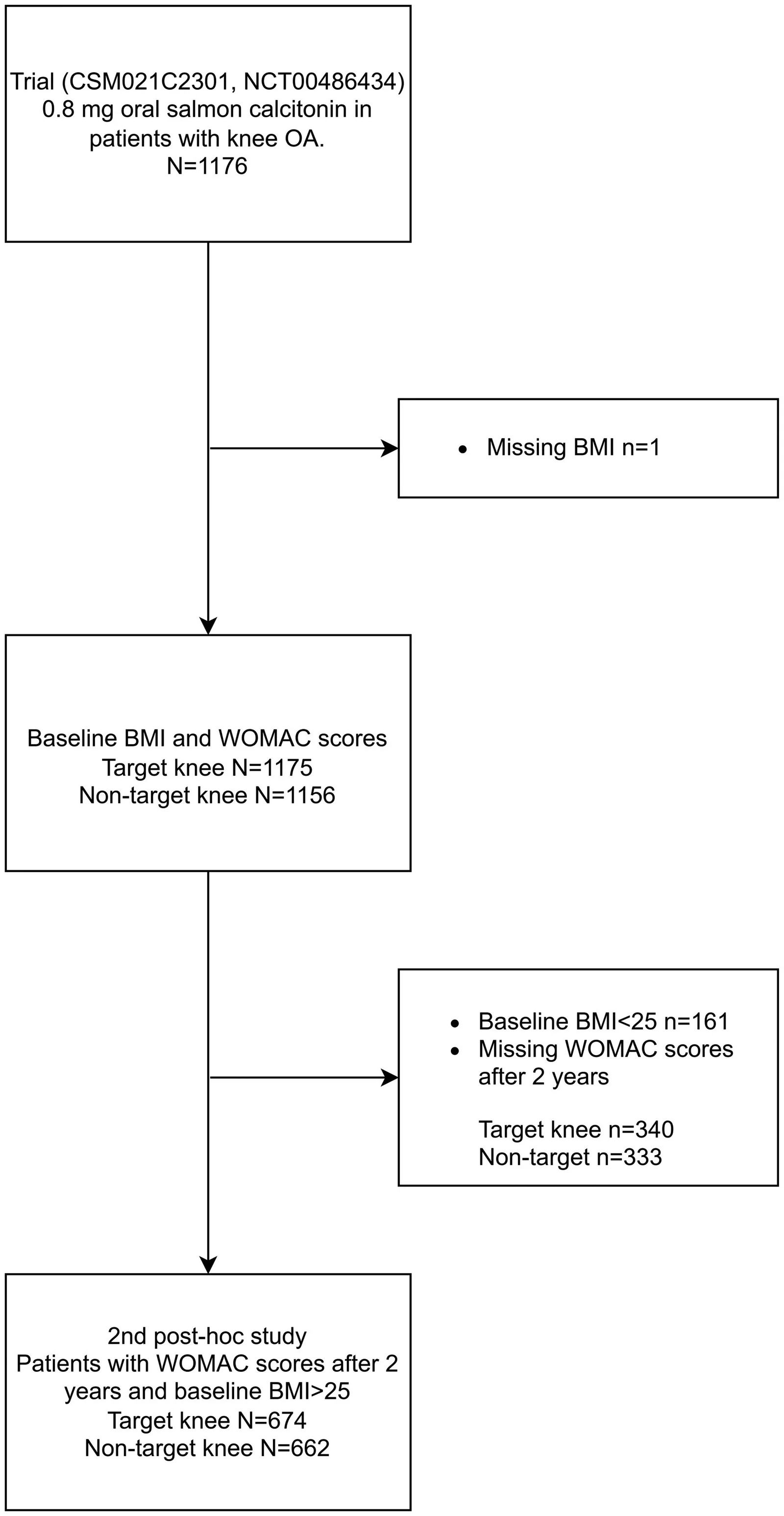
Study flow diagram. A total of 1176 patients were enrolled in the original study. Baseline BMI and WOMAC scores were analyzed for all patients. Post hoc analysis of the effect of weight loss on WOMAC scores after 2 years excluded patients with a BMI < 25 or missing WOMAC scores.

### Animal study

Male Sprague Dawley rats 7 to 8 weeks old (*n* = 32, Taconic, Denmark) were used. Animals were housed on a 12-hour light-dark cycle under 21 to 23 °C and 55% to 65% relative humidity in standard type IV cages, enriched with standard wood chips, red-tinted huts, nesting material, and sticks. Animals had ad libitum access to food and water. Sprague Dawley rats were divided into 2 groups (*n* = 16) and fed either a HFD constituting a 60 kcal% fat (D12492, OpenSourceDiets, Denmark) or a normal chow diet (ND; 1328, Altromin, Germany) for 3 months before OA induction. All animal procedures were performed following guidelines from the Animal Welfare inspectorate of the Danish Ministry of Justice under the institutional license issued to Nordic Bioscience (2015-15-0201-00469).

### Surgical OA induction

Osteoarthritis was induced surgically as previously described (15, 16). Briefly, isoflurane was used to anesthetize rats, and bupivacaine was injected locally for local anesthesia. After shaving and disinfecting the right leg, the medial collateral ligament was cut, and the medial meniscus was transected and removed. Sham animals went through the same procedure without affecting the joint space or any joint structures. Carprofen 5 mg/kg every 24 hours was subcutaneously administered for 3 days as postoperative analgesia. Meniscectomy model establishment was done on 12 rats split into 2 groups; 6 underwent meniscectomy (MNX) surgery, and 6 received Sham surgery. While in the metabolic model study, after 3 months on HFD, group allocation was done randomly according to their body weights: ND-Sham (*n* = 8), ND-MNX (*n* = 8), HFD-Sham (*n* = 8), and HFD-MNX (*n* = 8).

### Von Frey test

Mechanical allodynia was assessed by measuring 50% paw-withdrawal thresholds (PWTs) in the ipsilateral paw (right paw). Paw-withdrawal threshold was quantified by applying von Frey filaments according to the previously explained up–down method (17). After habituation and training, PWT was measured 2 weeks after arrival and followed for 3 months until OA induction, then once a week for 4 weeks after surgery by a blinded experimenter, in groups randomly assigned and representative of each condition.

### Cold hyperalgesia

Cold hyperalgesia was assessed using a cold plate (Hot/Cold Plate 35150, Ugo Basile SRL, Italy). Animals acclimatized to the cold plate in the absence of cold stimulus twice before collecting baseline measurements. Each measurement involved 5 minutes of exposure for each animal on the plate at 5 °C, which was filmed with a video camera. The test was repeated once a week for 4 weeks after surgery, in groups randomly assigned and representative of each condition. The filmed videos were used to determine each animal's score, blinded to intervention groups, by counting paw lifts of each paw and computing the paw-lift score (paw-lift score = ipsilateral paw lifts − contralateral paw lifts). A paw-lift counts when the animal lifts or flicks 1 paw clearly; walking or jumping does not count as paw lifts (18).

### Histology

Eleven weeks after OA induction, animals were euthanized by isoflurane anesthesia followed by exsanguination. The ipsilateral knees were collected, fixed in 4% formaldehyde for 3 days, and then decalcified in 15% EDTA buffer for 2 months. Knee joints were embedded in paraffin after being processed in Tissue-Tek VIP 5 Jr. (Sakura Finetek, Alphen aan den Rijn, The Netherlands). Paraffin blocks were cut into 5 µm coronal sections using an HM 360 microtome (Microm International GmbH, Walldorf, Germany). After deparaffinization and rehydration, sections were stained with toluidine blue stain (Merch KGaA, Darmstadt, Germany), as previously described (19). Sections were scored according to the Osteoarthritis Research Society International (OARSI) OA pathology assessment system (20). The depth of damage and pathologies determine OA's grade, while the area affected provides the OA stage. The score given is a product of OA's grade and stage (OA score = grade × stage). Two blinded personnel scored 5 sections per animal involving the medial tibial plateau, reporting the average score.

### Statistical analysis

Data from placebo and oral salmon calcitonin treatment groups were used in this post hoc analysis. Baseline differences between BMI categories were analyzed by analysis of covariance (ANCOVA), adjusting for age, sex, and BMI and reporting Bonferroni-corrected significance levels (*P*-values). Data of patients with complete WOMAC scores after 2 years and had a BMI > 25 were used in the analysis of the effect of weight loss on changes in WOMAC scores after 2 years using ANCOVA, adjusting for age, sex, BMI, and baseline WOMAC score/subscore, and reporting Bonferroni-corrected significance levels (*P*-values). Baseline WOMAC scores for included and excluded patients in the post hoc analysis were compared using ANCOVA.

In the preclinical study, a 2-way analysis of variance with Tukey multiple comparisons adjustment was used to compare the means of the net area under the curve across groups, assuming normality. Von Frey test data were normalized as percentage change from baseline. The sample size was decided based on previous studies on the MNX model (16). The data shown in the text and plots represent the mean with 95% CIs. Robust regression and outlier removal (ROUT) test was used to identify outliers with a false discovery rate (*Q* = 1%). The significance level was set at *P* < .05 (2-tailed). GraphPad Prism 9.1 was used to generate plots (Graph Pad Software, La Jolla, CA, United States).

## Results

### Baseline association between BMI and WOMAC scores

We compared the baseline WOMAC scores of 1175 patients across different BMI categories included in our first post hoc analysis, as outlined in Fig. 1 and Table 1. The mean age across the 4 groups was comparable. Females represented the majority of all the groups. The KL grade distribution in the target knees was similar across the 4 groups. Both target and nontarget knee WOMAC scores showed significantly higher mean baseline total and function scores in the BMI > 35 kg/m^2^ group than in the BMI < 25 kg/m^2^ group (Table 1). The nontarget knee mean baseline WOMAC pain score was higher in the BMI > 35 kg/m^2^ group than in the BMI < 25 kg/m^2^ group, in addition to a higher mean function score than in the BMI 25 to 30 kg/m^2^ group (Table 1). Overall, higher BMI is associated with higher WOMAC scores.

**Table 1:** Baseline characteristics of patients

	All participants	<25 kg/m^2^	25-30 kg/m^2^	30-35 kg/m^2^	>35 kg/m^2^
Target knees, ***n***	1175	226	526	302	121
Nontarget knees, *n*	1156	226	517	299	114
Age, years	64 (59.6, 69.1)	64.9 (59.9, 68.7)	64.7 (59.7, 69.2)	64.8 (59.6, 69.6)	63.2 (58.5, 67.4)
Sex, female, ***n* (%)**	804 (68.4)	172 (75.8)	320 (60.8)	215 (71.2)	97 (80.2)
Race, *n* (%)					
Asian	104 (8.8)	49 (21.6)	47 (8.9)	8 (2.6)	0
White	1071 (91.1)	178 (78.4)	478 (90.9)	294 (97.4)	121 (100)
Other	1 (0.09)	0	1 (0.2)	0	0
BMI, kg/m^2^	28.4 (25.7, 31.6)	23.4 (22.5, 24.3)	27.5 (26.2, 28.8)	32 (30.9, 33.4)	37.5 (35.9, 39.5)
Kellgren–Lawrence grade					
Target, *n* (%)					
2	1032 (87.8)	208 (91.6)	464 (88.2)	256 (84.8)	104 (86)
3	144 (12.2)	19 (8.4)	62 (11.8)	46 (15.2)	17 (14)
Kellgren–Lawrence grade					
Nontarget, *n* (%)					
0	36 (3.1)	6 (2.7)	20 (3.9)	6 (2)	4 (3.4)
1	229 (20.0)	56 (24.9)	110 (21)	49 (16.6)	14 (12.7)
2	633 (55.3)	133 (59.1)	281 (54.7)	170 (57.4)	49 (44.5)
3	215 (18.8)	28 (12.4)	87 (16.9)	61 (20.6)	39 (35.5)
4	32 (2.8)	2 (0.9)	16 (3.1)	10 (3.4)	4 (3.6)
Target knee WOMAC score					
WOMAC total, 2400 points	1097 (851, 1395.8)	1027 (830, 1342)	1070.5 (848, 1392)	1180.5 (866, 1420)**	1201 (928.3, 1491.5)**
WOMAC function, 1700 points	781 (595.3, 1006.5)	735.5 (557, 961)	757 (585, 994)	836 (617, 1021)*	845 (639.5, 1077)**
WOMAC pain, 500 points	228.5 (179, 286.5)	220 (172.3, 274.5)	228.5 (180, 284)	232 (189, 291)	239 (180.8, 287)
WOMAC stiffness, 200 points	100 (65, 131)	93 (57, 129.8)	99 (66, 128)	104 (67, 134)	104 (64.3, 135)
Nontarget knee WOMAC score					
WOMAC total, 2400 points	888 (486.5, 1280)	798 (474, 1165)	880 (432, 1275)	916 (535, 1309)*	1061 (625, 1479)***
WOMAC function, 1700 points	640 (323, 931)	566 (306, 867)	627 (302.8, 922.3)	659 (393.8, 974.8)*	751 (448, 1066)***
WOMAC pain, 500 points	173 (90, 253.3)	159 (80, 239)	173 (83.8, 257.3)	177 (105, 246.8)	199 (110, 286)*
WOMAC stiffness, 200 points	75 (32, 117)	71 (29.5, 105.8)	74 (27.8, 115)	76 (34, 120.8)	94 (46, 135)*

Data presented as median (Q25, Q75) or number (percentage).

^*^Statistical significance: *P* < .05.

^**^Statistical significance: *P* < .01.

^***^Statistical significance: *P* < .001 vs <25 kg/m^2^ using ANCOVA test.

### Baseline characteristics of patients included in the weight loss effect on WOMAC scores

A total of 674 patients completed their 2-year follow-up and were included in the analysis, with a mean age of 64.3 years, predominantly female (64.2%), and a mean BMI of 29.5 kg/m^2^. The analysis was conducted separately for the patients’ target and nontarget knees. The target knee population had a median (interquartile range) baseline WOMAC total, function, pain, and stiffness scores of 1125.5 (871-1395), 799.5 (611-1007), 230.5 (184-285), and 103 (70-132), respectively, and the nontarget knee had a median baseline WOMAC total, function, pain, and stiffness scores of 875 (463-1283), 628 (308-940), 172 (83-251), and 76 (30-117), respectively (Table 2). Baseline characteristics of patients excluded from the study due to missing WOMAC scores at 2-year follow-up or because they had a BMI < 25 are presented in Table S1 (21) and show similar baseline values.

**Table 2:** Baseline characteristics of patients included in the post hoc analysis of the effect of weight loss on WOMAC scores

	All participants	>5% weight loss	≤5% weight loss or gain	>5% weight gain
Target knees, *n*	674	100	518	56
Nontarget knees, *n*	662	98	510	54
Age, years	64.3 (59.6, 69.1)	64 (60, 69.8)	64.6 (59.8, 69.1)	62.5 (58.1, 67.4)
Sex, female, *n* (%)	433 (64.2)	71 (71)	325 (62.7)	37 (66.1)
Race, *n* (%)				
Asian	42 (6.2)	6 (6)	36 (6.9)	0
White	631 (93.6)	94 (94)	481 (92.9)	56 (100)
Other	1 (0.1)	0	1 (0.2)	0
BMI, kg/m^2^	29.5 (27.3, 32.6)	30.3 (27.8, 34.1)	29.3 (27.3, 32)	30 (27.4, 33.7)
Treatment, *n* (%)				
Placebo	350 (51.9)	44 (44)	279 (53.9)	27 (48.2)
Salmon Calcitonin (sCT)	324 (48.1)	56 (56)	239 (46.1)	29 (51.8)
Kellgren–Lawrence grade				
Target, *n* (%)				
2	580 (86.1)	81 (81)	448 (86.5)	51 (91.1)
3	94 (13.9)	19 (19)	70 (13.5)	5 (8.9)
Kellgren–Lawrence grade				
Nontarget, *n* (%)				
0	19 (2.8)	2 (2.1)	14 (2.7)	3 (5.5)
1	117 (17.5)	12 (12.4)	95 (18.4)	10 (18.2)
2	376 (56.4)	56 (57.7)	297 (57.7)	23 (41.8)
3	133 (19.9)	22 (22.7)	97 (18.8)	14 (25.5)
4	22 (3.3)	5 (5.2)	12 (2.3)	5 (9.1)
Target knee WOMAC score				
WOMAC total, 2400 points	1125.5 (871, 1395)	1084 (849.5, 1373.5)	1121.5 (871, 1394)	1247.5 (961.5, 1418)
WOMAC function, 1700 points	799.5 (611, 1007)	779.5 (617, 1000.5)	789 (605, 1010)	882 (675, 1006)
WOMAC pain, 500 points	230.5 (184, 285)	217.5 (175.5, 284)	232.5 (184, 284)	246.5 (194, 304)
WOMAC stiffness, 200 points	103 (70, 132)	100 (57, 135)	103 (71, 132)	110 (82, 132.5)
Nontarget knee WOMAC score				
WOMAC total, 2400 points	875 (463, 1283)	890.5 (570, 1366)	862.5 (424, 1248)	1057.5 (532, 1382)
WOMAC function, 1700 points	628 (308, 940)	697.5 (413, 982)	614.5 (294, 916)	745.5 (298, 1000)
WOMAC pain, 500 points	172 (83, 251)	179 (110, 265)	168.5 (80, 245)	194.5 (112, 261)
WOMAC stiffness, 200 points	76 (30, 117)	76 (32, 115)	74 (30, 116)	92.5 (26, 123)

Data presented as median (Q25, Q75) or number (percentage).

### Weight loss is associated with lower pain scores

The WOMAC scores after 2 years for the target knee were associated with a significantly greater reduction in mean WOMAC total and function scores in the >5% weight loss group −567.1 (95% CI −666.7 to −467.6), −403.7 (−477 to −330.4) compared with the >5% weight gain group −364.6 (−496.5 to −232.7), −247.2 (−344.4 to −150.1), respectively, (Fig. 2A-2D). The nontarget knee were associated with considerably larger reductions in the mean total and function WOMAC scores after 2 years in both the >5% weight loss −399.2 (−505.8 to −292.5), −299.6 (−377.2 to −222) and the ≤5% weight loss or weight gain groups −245.2 (−291.5 to −199), −172 (−205.9 to −138.3), compared with patients in the >5% weight gain group −135 (−277.4 to −6.3), −87.3 (−191.1 to −16.4), respectively (Fig. 3A-3D).

**Figure 2:**
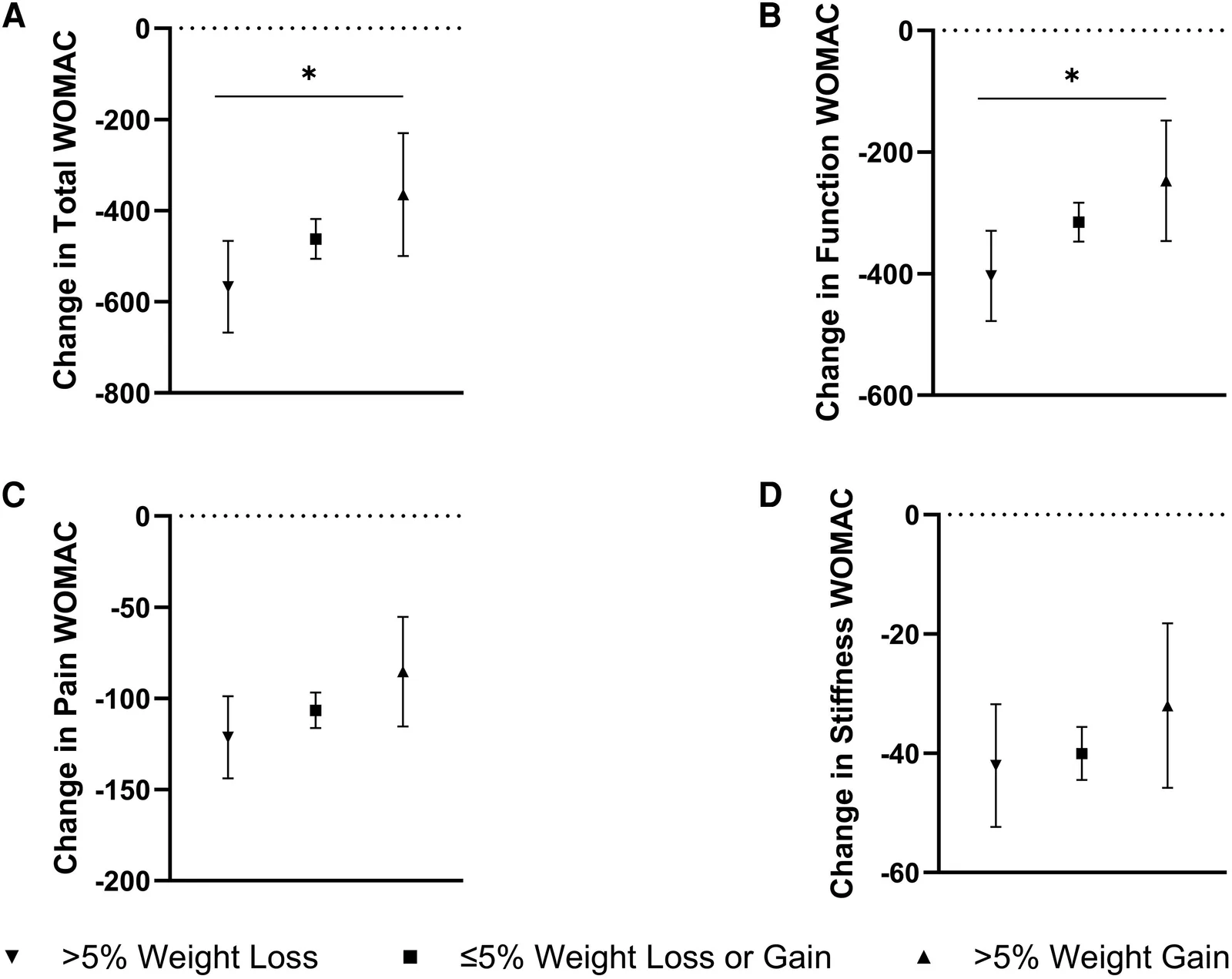
Changes in WOMAC scores in patients who lost or gained weight over 2 years for the target knee: (A) total, (B) functional, (C) pain, and (D) stiffness WOMAC scores. ANCOVA was used for analysis. Data presented as mean and error bars indicate 95% CIs. Statistical significance is indicated by *P < .05.

**Figure 3:**
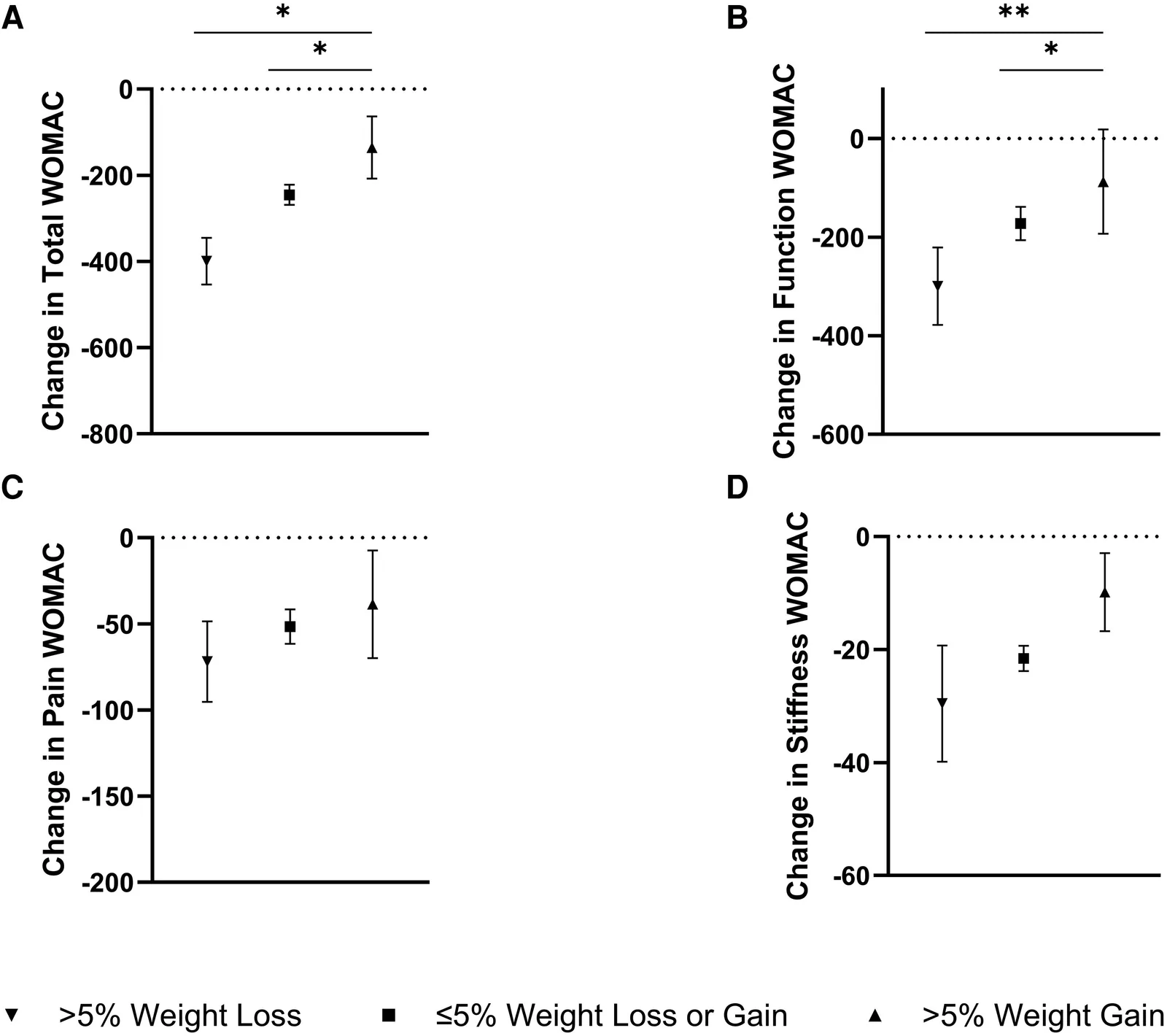
Changes in WOMAC scores in patients who lost or gained weight over 2 years for the nontarget knee: (A) total, (B) functional, (C) pain, and (D) stiffness WOMAC scores. ANCOVA was used for analysis. Data presented as mean and error bars indicate 95% CIs. Statistical significance is indicated by *P < .05, **P < .01.

### HFD-induced obesity leads to increased sensitivity to mechanical and cold stimuli in rats

To investigate the causal relationship between obesity and OA pain, we examined pain-like symptoms in rats. Before OA induction, both HFD-fed rats and ND-fed rats increased in body weight, while the HFD group had put on more weight than the ND group (Fig. S1A) (21). After 2 weeks on HFD, HFD-fed rats exhibited a significantly lower mean PWT than the ND-fed rats. The lower PWT of the HFD group persisted until the 40th day. The following time points showed comparable PWT for both groups (Fig. S1B) (21).

High-fat diet and ND groups were randomized based on body weight and assigned to either MNX or Sham surgery (ie, 4 groups in total). After OA induction, the 4 groups were HFD-MNX, HFD-Sham, ND-MNX, and ND-Sham (*n* = 8 per group). The induction of OA in both the HFD-MNX and ND-MNX groups led to a significant reduction in the 50% PWT (log(*g*)) throughout the 30 days postsurgery (Fig. 4A and 4B). The cold plate test data did not show a significant difference when directly comparing the paw-lift scores of the MNX and non-MNX groups (Fig. 4C and 4D). However, an interaction between HFD and MNX surgery was found (*P* < .05).

**Figure 4:**
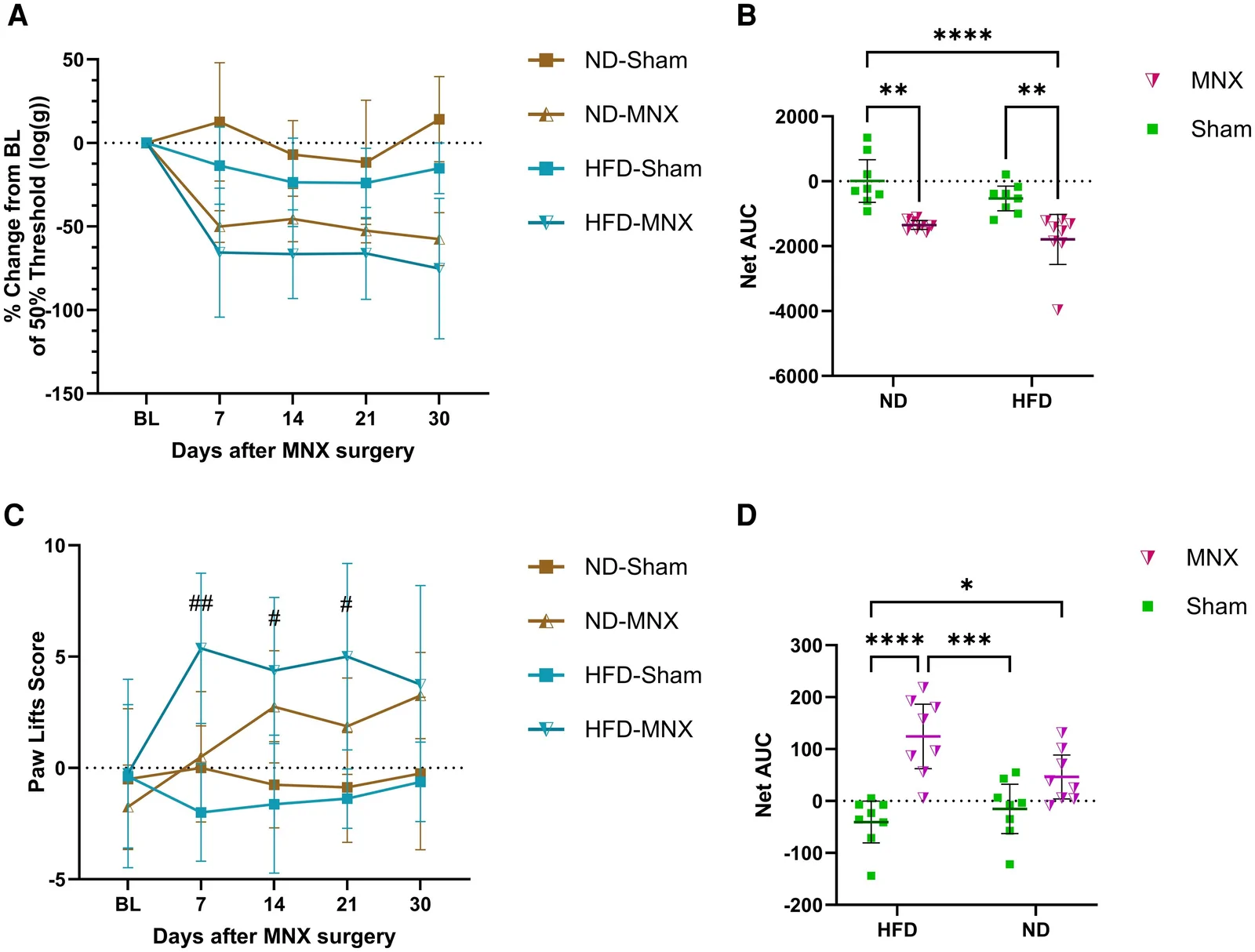
Obesity and OA induce sensitivity to mechanical and cold stimuli. (A) The mean percentage change from baseline of the 50% PWT of meniscectomized on a HFD (HFD-MNX), Sham on a HFD (HFD-Sham), meniscectomized on normal chow diet (ND-MNX), and Sham on normal chow diet (ND-Sham) groups. (B) The mean net area under the curve (AUC) derived from (A) for each animal. (C) The mean paw-lift score (contralateral paw-lifts subtracted from ipsilateral paw-lifts) of HFD-MNX, HFD-Sham, ND-MNX, and ND-Sham groups (# HFD-MNX vs HFD-Sham). (D) The mean net AUC derived from (C) for each animal. Two-way analysis of variance was used for analysis. Error bars indicate the 95% CI, *n* = 8/group. Statistical significance is indicated by *P < .05, # *P* < .05, **P < .01, ## *P* < .01, ***P < .001, and **** *P* < .0001.

### Effect of metabolic OA on joint structure

Compared to the ND-Sham group, representative toluidine blue-stained sections of the medial tibial cartilage indicated clear pathological signs of OA in the HFD-MNX and ND-MNX groups. The ND-Sham group showed intact articular cartilage with no detectable signs of damage. The HFD-Sham group exhibited some signs of damage, including excavation of the superficial and midzone. The ND-MNX group displayed hypertrophy, mid-layer cysts, and fibrocartilage. The HFD-MNX group showed destruction of the tidemark and replacement of the articular hyaline cartilage with fibrocartilage, in addition to features of bone remodeling, including subchondral bone thickening and deformation (Fig. S2A) (21). The meniscectomized animals showed significantly higher scores on both diets (*P* < .0001; Fig. S2B) (21). The statistical analysis has shown an interaction between HFD and structural deterioration (*P* < .01), but no differences were found comparing the individual groups.

## Discussion

Obesity is a common risk factor and a known driver of many debilitating diseases, including OA (22). With populations increasing in age and the rise in individuals with obesity, the prevalence of OA has been increasing worldwide (23). Chronic inflammation and joint overloading from obesity exacerbate articular cartilage damage, thereby speeding the progression of OA and aggravating pain (24). In this study, we investigated the relationship between obesity and joint pain/function using a translational approach. The post hoc analysis showed that patients with OA experience more intense OA pain when they belong to a higher BMI group than a lower BMI group. Osteoarthritis patients who lost >5% of body weight during the trial were associated with a greater reduction in WOMAC scores than those who did not. This finding is in line with previous studies investigating the effect of weight loss on OA pain (25, 26). However, in this study we evaluated both target and nontarget knees. Interestingly, the nontarget knee showed a more considerable association with improvement. Furthermore, in our metabolic animal model of OA, we examined the relationships among obesity, OA, and pain-like symptoms. The combination of HFD as a metabolic stressor and OA progression triggered by MNX provided a disease phenotype that incorporated the metabolic element, highlighting its effect on pain-like symptoms.

The post hoc analysis indicates a connection between osteoarthritic pain, function, and body weight. We observed that 5% or more weight loss was associated with lower reported pain scores, whereas other studies have indicated that greater weight loss is needed to impact joint pain and function (25-28). Additionally, the reduction in body weight was associated with lowered WOMAC pain scores in the nontarget knee across a broader range of KL grades. As a result of decreased mechanical load and potentially shrinking adipose tissues, this may modulate metabolically induced low-grade inflammation and central sensitization pain pathways (29, 30). However, the magnitude and rate of weight loss are crucial for reducing pain and modifying the disease trajectory, in addition to providing clinically meaningful reduction. A study showed that a 7% reduction within 35 weeks is significant enough to reduce OA pain (28). Despite this, rapid and intense weight loss is associated with negative consequences for muscular and skeletal health, which are critical for supporting the knee joint structure (31, 32). The loss in mass includes significant increases in bone and cartilage degradation biomarkers such as CTX-I and CTX-II (31). Overall, these data are in line with previous studies on the beneficial effects of weight loss on OA symptoms and, as such, point to weight as a factor in the development of novel therapies. Nevertheless, the magnitude, method, and rate of achieving weight loss remain fundamental in modifying the course of symptomatic experience and the progression of the disease.

To strengthen our understanding of the interplay between OA and obesity, we investigated the impact of obesity on pain sensation in a preclinical rat model combining HFD and surgically induced OA. We found that pain sensitivity increased temporarily with an HFD, which might be explained by increased systemic inflammation and rapid weight gain associated with obesity. Indeed, an increase in sensitivity to stimuli has been shown in previous publications (33-35). Here, this metabolic model showed signs of pain-like symptoms that match the pain observed in OA patients with a high body mass index, presenting with worse pain with increased BMI (36). This aligns with the increase in pressure sensitivity shown in people with obesity compared with healthy-weight people, underscoring the association between obesity and pain (37).

To further characterize the impact of obesity on pain sensation, we combined HFD with MNX to elicit joint damage and pain. In the metabolic model, animals with obesity underwent OA induction, after which pain-like symptoms were observed in both lean groups and groups with obesity using the von Frey and cold plate tests, compared with Sham groups. The results also indicated that the group with obesity had a lower threshold for pain sensation, and the data overall showed a significant effect of HFD on symptoms when comparing groups with obesity to lean groups. Previous studies investigated the effect of a corn starch diet or a high-carbohydrate HFD on an OA pain-like phenotype and found that both increased pain sensation (38). Their findings align with ours, suggesting that HFD worsens OA pain-like symptoms, particularly mechanical sensitivity to stimuli. Additionally, their findings show that different high-calorie diets generally induce similar effects on the nociceptive experience. Interestingly, this also aligns with clinical data from the literature, indicating that obesity increases pain sensitivity across the body and, more so, in OA (25-27).

At the joint level, the effects of obesity in the model were less clear; possibly because the MNX surgery masked the potential effects of obesity on the joint structure. Similarly, other studies conducted in rodents also did not find substantial effects on joint structure (39). Namely, observations by Sekar et al (38), indicated that a HFD resulted in cartilage damage in addition to a decrease in bone mineral density and also illustrate the difficulty in demonstrating very clear HFD-induced effects on joint integrity.

There are several limitations to the study. First, the clinical study was not designed or powered to investigate causal relationships between pain and obesity, making it challenging to find clinically meaningful differences and does not completely rule out confounders such as reverse causality. Secondly, the subjects in the cohort were recruited with strict inclusion and exclusion criteria and, as such, only represented a narrow pain span and a rather specific OA subtype (11). In the preclinical model, behavioral readouts show that the main disease driver is the trauma from the MNX, while obesity does worsen the condition; the balance is still skewed toward a traumatic inducer. The induction and progression of obesity and OA still do not accurately represent the course of the complex metabolic disease in humans. Also to be noted is that our study involved only male rats, and with a larger sample size and less variable outputs from behavioral tests, smaller differences might have been detectable. The unavailability of sensitive rodent biomarker assays made it challenging to connect functional and molecular readouts, and in future studies, it would be of great interest to favor the effects of obesity more.

In conclusion, we observed a connection between obesity and intensified OA pain. Our clinical data support the association between obesity and joint pain in OA. These findings also suggest that weight loss could reduce OA pain. Furthermore, adding a metabolic component to the MNX rat model demonstrated that obesity increases pain-like sensitivity, which supports the translatability of this metabolic OA model. Considering that obesity is one of the leading causes of OA, implementing models with obesity during drug development may facilitate the development of novel metabolically active drug candidates for OA, for example, incretins or amylin-based molecules.

## Data Availability

The data that support the findings of this manuscript are available from the corresponding author upon reasonable request.

## Clinical trial information

Trial registration number RCT (NCT00486434).

## Abbreviations

ANCOVA: analysis of covariance
HFD: high-fat diet
KL: Kellgren and Lawrence
MNX: meniscectomy
ND: normal diet
PWT: Paw-withdrawal threshold
WOMAC: Western Ontario and McMaster Universities Osteoarthritis index
